# Mechanism of hydrogen peroxide formation by lytic polysaccharide monooxygenase†
†Electronic supplementary information (ESI) available. See DOI: 10.1039/c8sc03980a


**DOI:** 10.1039/c8sc03980a

**Published:** 2018-10-19

**Authors:** Octav Caldararu, Esko Oksanen, Ulf Ryde, Erik D. Hedegård

**Affiliations:** a Division of Theoretical Chemistry , Lund University , Chemical Centre , P. O. Box 124 , SE-221 00 Lund , Sweden . Email: octav.caldararu@teokem.lu.se ; Email: erik.hedegard@teokem.lu.se; b European Spallation Source ESS ERIC , P. O. Box 176 , SE-221 00 Lund , Sweden; c Department of Biochemistry and Structural Biology , Lund University , Chemical Centre , P. O. Box 124 , SE-221 00 Lund , Sweden

## Abstract

Lytic polysaccharide monooxygenases (LPMOs) are copper-containing metalloenzymes that can cleave the glycosidic link in polysaccharides. This could become crucial for production of energy-efficient biofuels from recalcitrant polysaccharides. Although LPMOs are considered oxygenases, recent investigations have shown that H_2_O_2_ can also act as a co-substrate for LPMOs. Intriguingly, LPMOs generate H_2_O_2_ in the absence of a polysaccharide substrate. Here, we elucidate a new mechanism for H_2_O_2_ generation starting from an AA10-LPMO crystal structure with an oxygen species bound, using QM/MM calculations. The reduction level and protonation state of this oxygen-bound intermediate has been unclear. However, this information is crucial to the mechanism. We therefore investigate the oxygen-bound intermediate with quantum refinement (crystallographic refinement enhanced with QM calculations), against both X-ray and neutron data. Quantum refinement calculations suggest a Cu(ii)–O–2 system in the active site of the AA10-LPMO and a neutral protonated –NH_2_ state for the terminal nitrogen atom, the latter in contrast to the original interpretation. Our QM/MM calculations show that H_2_O_2_ generation is possible only from a Cu(i) center and that the most favourable reaction pathway is to involve a nearby glutamate residue, adding two electrons and two protons to the Cu(ii)–O–2 system, followed by dissociation of H_2_O_2_.

## Abstract 2

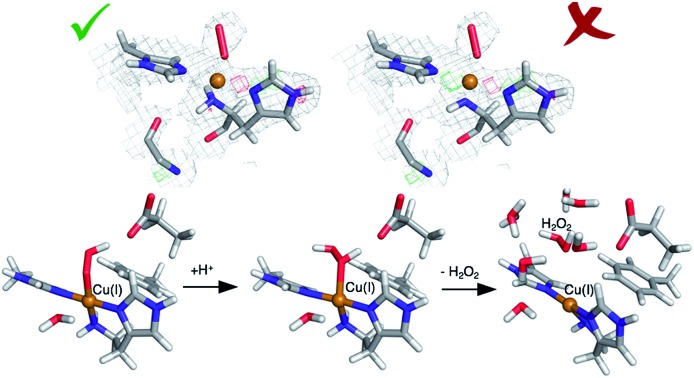
A mechanism for the formation of hydrogen peroxide by lytic polysaccharide monooxygenases (LPMOs) in the absence of substrate is proposed.

## 1 Introduction

Lytic polysaccharide monoxoygenases (LPMOs) are metalloenzymes capable of activating molecular oxygen, and thereby inserting a single oxygen atom into the C–H bonds that comprise the glycoside link in polysaccharides.^1^,^2^ This oxidation leads to cleavage of the glycoside link, which may disrupt the surface of crystalline polysaccharides sufficiently to boost polysaccharide decomposition.^3^ Hitherto, this decomposition has been a major obstacle for energy-efficient production of biofuels^4^–^6^ from recalcitrant polysaccharides such as cellulose, which is one of the most abundant polysaccharides on earth.^7^

**Fig. 1:**
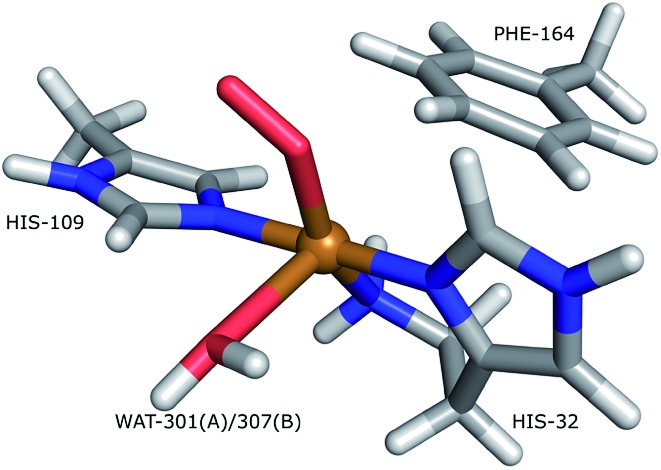
The active site of AA10-LPMOs with the histidine brace and the nearby Phe164 residue, numbering according to PDB 5VG0. The figure also shows to the employed quantum system in the quantum-refinement calculations. (A) and (B) refer to chain A and B, respectively.

A number of different LPMOs have been categorized, belonging to four distinct classes, AA9,^1^ AA10,^8^ AA11,^9^ and AA13.^10^,^11^ The various LPMOs have remarkably varying amino-acid sequences (no residues besides the histidine brace are strictly conserved between AA9, AA10, AA11 and AA13 LPMO classes) and target a wide range of different polysaccharide substrates.^12^–^15^ However, all LPMOs contain a copper ion^8^ coordinated by two histidine residues in what has become known as the histidine brace (*cf.*Fig. 1).^8^ In the histidine brace, one histidine is the amino-terminal residue that coordinates bidentately with both the N-terminus and the imidazole side chain.

It is believed that the mechanism of the LPMOs is initiated by reduction of Cu(ii) to Cu(i), followed by binding of O_2_ to Cu(i) to form a superoxide complex, [CuO_2_]^+^.

1 Cu^2+^ + e^–^ → Cu^+^

2 Cu^+^ + O_2_ → [CuO_2_]^+^

This complex has been suggested to be the reactive intermediate.^16^ However, theoretical work has shown that the superoxide is not sufficiently reactive to abstract a hydrogen from the glycoside C–H bond^17^,^18^ and points rather to either a copper-oxyl,^17^–^21^ [CuO]^+^, or a copper-hydroxy,^18^,^21^ [CuOH]^2+^ species. These more reactive copper–oxygen species can be generated by successive reductions and protonations of the superoxide species.^4^–^6^,^22^,^23^

Another issue concerns the co-substrate. Bissaro *et al.*^24^ recently suggested that H_2_O_2_, rather than O_2_, is the actual co-substrate, whereas Hangasky *et al.* proposed that both O_2_ and H_2_O_2_ can be utilized by LPMOs as co-substrates.^25^ Intriguingly, LPMOs in fact generate hydrogen peroxide in absence of a polysaccharide substrate.^12^

In a landmark study, combining theoretical and experimental methods, Kjærgaard *et al.*^26^ investigated the initial reduction and reaction with O_2_ (eqn (1) and (2)). Their data showed that Cu(ii) and O_2_ form a superoxide [CuO_2_]^+^ species, which is rapidly displaced by water to regenerate the resting state. Based on DFT calculations, they further suggested a mechanism for superoxide release by concerted O–2 dissociation and protonation of O–2 to HO_2_ by an axially coordinated water molecule. This is a feasible mechanism for H_2_O_2_ formation, as HO_2_ is known to form H_2_O_2_ through disproportionation in aqueous solution. However, no second-sphere residues were taken into account as possible proton donors.

Regarding the oxygen species, the paper by Kjærgaard *et al.*^26^ is today supported by the X-ray and neutron diffraction study by O'Dell *et al.*,^27^ which shows an O_2_-bound intermediate from an AA9 LPMO, although they interpreted the species as a peroxide-bound (O2–2) intermediate. More recently, a similar intermediate was trapped in a crystal structure of an AA10 LPMO,^28^ investigated by both X-ray and neutron diffraction. This study has further attracted attention since the protein crystallizes as a dimer of subunits A and B, where the amino-terminal atom seems to be a mixture of –ND^–^ and –ND_2_ in subunit B (but a pure –ND_2_ state in subunit A). The interpretation is based on an asymmetric nuclear difference density peak in subunit B, which can be attributed to a partially occupied site for the D atom. Deprotonation of the N-terminus in LPMOs has been suggested previously, partly from studies of model complexes^29^ and partly based on the fact that the chemical environments of the two protons are different for substrate-bound LPMO.^30^,^31^ It is possible that the N-terminus has increased acidity, due to coordination to the Cu ion and possibly also due to a hydrogen-bonding network generated upon substrate binding,^31^ but it should be remembered that a free (not metal-bound) N-terminus is normally triply protonated and positively charged (–NH+3 with a p*K*_a_ of ∼8) in water solution.

Thus, CuO_2_ or [CuO_2_]^+^ species are most likely not active for C–H abstraction, but they may very well be relevant for H_2_O_2_ generation. However, the exact nature of the species (superoxide or peroxide) is unclear and the recent X-ray and neutron diffraction studies do not allow unequivocal assignment of the N-terminal protonation state (NH_2_ or NH^–^). The mechanism of peroxide generation cannot be fully investigated before this is clear. Therefore, the purpose of this study is two-fold. Our main goal is to investigate the mechanism of H_2_O_2_ generation, taking into account a nearby proton-donor residue that was previously neglected. Since the study of H_2_O_2_ generation depends on the nature of the copper–oxygen species, we also investigate the nature and coordination of this species in more detail. This is particularly pertinent, given that both the metal ion and the coordinated oxygen species are susceptible to radiation damage, making conclusions from X-ray structures dubious. Indeed, this is a known issue even in high resolution X-ray structures^32^ and has also previously been a problem in crystal structures for LPMOs.^33^,^34^ The problem can be partly remedied by a combination of quantum chemical methods and crystallographic refinement in what is known as quantum refinement.^35^ This method has been successfully used^36^,^37^ within X-ray crystallography to resolve issues with poor density around metal atoms. The N-terminal protonation state suggested from the neutron diffraction data of ref. 28 is also not entirely unambiguous. Interpreting nuclear scattering length density maps from refinements against only neutron data have proven quite difficult for several reasons. First, neutron data usually has a lower resolution than X-ray data, since including hydrogen atoms introduces many more parameters, so the data-to-parameter ratio is low. Second, if hydrogen and deuterium atoms are close to each other, the deuterium and hydrogen signals can cancel out, as they have opposite signs. Similar to X-ray crystallography, the local structure around critical sites can be improved by quantum refinement. Quantum refinement for neutron crystallography is still in its infancy and only a proof-of-principle study^38^ has been undertaken so far. Here we employ for the first time quantum mechanics to facilitate both the refinement of X-ray and neutron diffraction. The result is the most probable interpretation of the LPMO copper–oxygen species, and from these results we suggest a new mechanism for generation of hydrogen peroxide.

## 2 Methods

### Joint X-ray–neutron refinement

The low data-to-parameter ratio in neutron crystallography can often be significantly improved by refining against both X-ray and neutron data at the same time (joint X-ray–neutron refinement).^39^ Unfortunately, such a procedure can be complicated when there are differences between the reported neutron and X-ray structures as is the case in the structure reported by Bacik *et al.*,^28^ where the backbones of the two protein models do not perfectly superpose. This is presumably why no joint refinement was performed for the deposited neutron structure (entry ; 5VG1 (ref. 28)). However, the space groups of the X-ray and neutron crystals are identical and the unit cell parameters are very similar, suggesting that the two data sets are suited for joint X-ray–neutron refinement. We therefore decided to redo the refinement as a joint refinement. X-ray structure factors, coordinates, occupation numbers, *B* factors (entry ; 5VG0 (ref. 28)), as well as neutron structure factors (entry ; 5VG1 (ref. 28)) were obtained from the Brookhaven Protein Data Bank (PDB).^40^ Joint X-ray–neutron refinement was performed in *Phenix*^39^,^41^ using the phenix.refinement module and the coordinates from the X-ray model. Deuterium atoms were added at exchangeable sites and H atoms at the rest of the sites with the phenix.ready_set module. Deuterium atoms at the N-terminus were added manually.

### Quantum refinement

Crystallographic refinement is a global pseudo-energy minimization using an energy function of the form

3 *E*_cryst_ = *w*_X_*E*_Xray_ + *E*_MM_

or

4 *E*_cryst_ = *w*_X_*E*_Xray_ + *w*_N_*E*_neutron_ + *E*_MM_

for pure X-ray refinement and joint X-ray and neutron refinement, respectively. Here *E*_MM_ is an MM (or another empirical or statistical) energy function of the protein model, whereas *E*_Xray_ and *E*_neutron_ are target functions describing how close the current model reproduces the experimental X-ray and neutron data, respectively. We have used a joint maximum-likelihood target function.^42^ The two weight factors *w*_X_ and *w*_N_ are needed because *E*_MM_ typically is in energy units, whereas the other two terms are unit-less pseudo-energies.

For quantum refinement, *E*_QM/MM_ replaces *E*_MM_, meaning that QM replaces MM for a restricted, but interesting, part of the protein (system 1). In this work we employ

5 *E*_QM/MM_ = *E*_QM_1__ + *E*_MM_ – *E*_MM_1__

where the *E*_QM_1__ is the energy of the system described by QM, which we here denote system 1. *E*_MM_1__ is the MM energy of the same system. The remaining system is denoted system 2. Technical parameters associated with the QM/MM calculations are described in the next subsection.

The quantum refinement method is implemented in the ComQum-X software,^35^ which combines Turbomole with the Crystallography and NMR system (CNS),^42^ replacing *E*_MM_ in eqn (1) with *E*_QM/MM_ in eqn (2) to give our combined energy function

6 *E*_ComQum-X_ = *w*_X_*E*_Xray_ + *E*_QM_1__ + *w*_MM_ (*E*_MM_ – *E*_MM_1__)

In eqn (6), an additional scaling factor, *w*_MM_, was introduced because the statistics-based force field in CNS typically gives energies that are ∼3 times larger than an energy-based force field (*i.e. w*_MM_ is set to 1/3).

The quantum-refinement calculations were performed starting from the joint X-ray–neutron refined coordinates, *B* factors and occupancies. However, CNS does not support anisotropic *B* factors, so those were ignored. The quantum system used for all refinements is shown in Fig. 1. It consisted of the copper ion, the imidazole ring of His-109, the phenyl ring of Phe-164, the full His-32 residue, which coordinates to Cu through the terminal amino group and a crystal water molecule that coordinates to the copper atom. For the investigation of the protonation of the N-terminus, we ran quantum refinement calculations including the neutron data. We can include neutron data in eqn (6) simply by adding a *E*_neutron_ term and the corresponding weight factor, the equation thus becoming

7 *E*_ComQum-U_ = *w*_X_*E*_Xray_ + *w*_N_*E*_neutron_ + *E*_QM_1__ + *w*_MM_ (*E*_MM_ – *E*_MM_1__)

This has recently been implemented in the ComQum-U software,^38^ combining Turbomole with the joint X-ray–neutron refinement version of CNS, nCNS. We assumed that the total charge of the quantum system was 0, *i.e.* corresponding to a Cu(ii)–peroxide system (CuO_2_) as in ref. 27. In a separate set of calculations, we exclusively employed quantum refinement of the X-ray diffraction data in ref. 28, and in this case we investigated both CuO_2_ and [CuO_2_]^+^ forms. The full protein was used in all calculations, including all crystal water molecules. In each cycle of the geometry optimization, the surrounding protein was allowed to relax by one cycle of crystallographic minimization and one cycle of individual *B* factor refinement. However, the new coordinates and *B* factors were accepted only if the *R* factor was reduced. After quantum-refinement, anisotropic *B* factor and occupancy refinement was performed using phenix.refine. All refinements were done at the TPSS/def2-SV(P) level of theory. Separate refinements were run with the quantum system in subunit A and subunit B respectively.

### Model quality metrics

The quality of the models was compared using the real-space difference density *Z*-score (RSZD) per atom, calculated by EDSTATS in the CCP4 software suite,^43^ which measures the local accuracy of the model. The maximum absolute value of RSZD is typically <3.0 for a good model. RSZD– shows the maximum negative RSZD value, whereas RSZD+ shows the maximum positive RSZD value.

### QM/MM calculations

QM/MM calculations were performed with the ComQum interface,^44^,^45^ which combines the QM program Turbomole and the MM program AMBER. Here we employed Turbomole 7.1 (ref. 46) and AMBER 14,^47^ respectively. When there is a bond between systems 1 and 2 (a junction), the hydrogen link-atom approach is employed: the QM region is capped with hydrogen atoms (hydrogen link atoms), the positions of which are linearly related to those of the corresponding carbon atoms (carbon link atoms) in the full system.^44^,^48^

The chosen QM method was exclusively density functional theory using the dispersion-corrected TPSS-D3 functional.^49^,^50^ We performed two sets of QM/MM calculations. The first was carried out as comparison to the quantum refinement. Here the QM systems (system 1) were designed to be identical (*i.e.* the one in Fig. 1), and we employed the def2-SV(P) basis set.^51^ We again employed total charges of the quantum systems of either +1 ([CuO_2_]^+^) or 0 (CuO_2_). In the former model, this involves Cu(ii) coupled to a superoxide anion, O–2, whereas the latter model involves either Cu(i) and O–2 or Cu(ii) and peroxide O2–2 (a detailed analysis of the wave function is required to determine which of the two formulations is the most appropriate). Note that while CuO_2_ was exclusively considered in the doublet spin-state, the [CuO_2_]^+^ moiety can, from previous experience,^17^,^21^,^52^ result in a triplet or an (open-shell) singlet state that are nearly degenerate in energy, and we therefore considered both cases. Separate calculations were performed with the quantum system in subunit A or B, respectively.

In a second set of calculations, we studied the mechanism of H_2_O_2_ generation. Here we extended the QM system with the side chain of the Glu-201 residue, which is a possible proton donor in the vicinity of the active site. In a some of the calculations, six additional water molecules close to the active site were added to the quantum system. These studies were performed only on subunit B, as this showed less variation in the binding of the oxygen species. The studies of proton donation and dissociation of the resulting species exclusively employed the def2-TZVPD basis sets.^51^,^53^

A more detailed account of the computational and protein setup (including the alternate configurations and protonation states of individual amino acids) are provided in the ESI.†

## 3 Results and discussion

In this paper, we discuss two important aspects of the LPMO copper center. First, we re-investigate the nature of the Cu–oxygen species observed in crystal structures, both in terms of oxidation state and protonation state of the terminal amino group. This is done by carrying out a joint refinement of the X-ray and neutron diffraction data reported in ref. 28. Moreover, we employ quantum refinement against both neutron and X-ray data. Second, we suggest a new mechanism of H_2_O_2_ formation, based on QM/MM calculations starting from the oxygenated species we deem most probable from the quantum refinements.

### Joint X-ray–neutron refinement

The joint X-ray–neutron crystallographic refinement employed the X-ray coordinates (entry 5VG0) as starting coordinates and we first considered the N-terminus in both subunits as ND_2_. The model was refined to X-ray *R*_free_ and *R*_work_ values of 14.4% and 13.9% respectively, and neutron *R*_free_ and *R*_work_ values of 24.8% and 19.2%. These values are comparable to those for the models deposited in the PDB (X-ray *R*_free_ of 12.8% and neutron *R*_free_ of 26.5%), showing an improvement of the model quality for the neutron structure but a slightly worse X-ray model. This is generally expected from a joint refinement.^39^

**Fig. 2:**
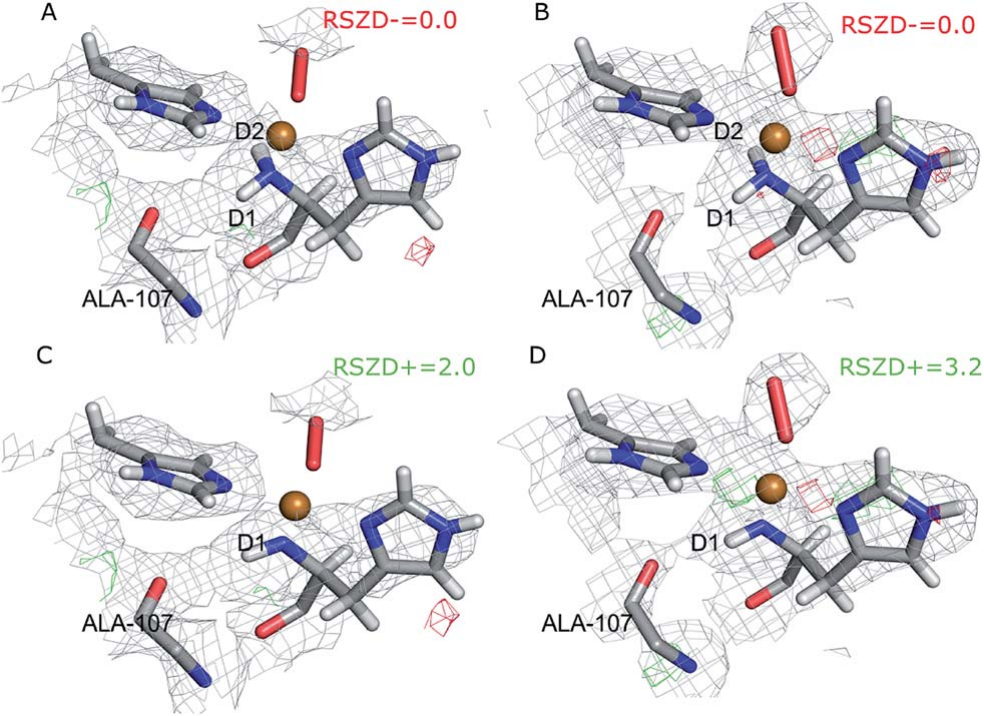
Structure and nuclear density maps of the active site after joint refinement. The *m*2*F*_o_ – *DF*_c_ maps are contoured at 1.0*σ* and the *mF*_o_ – *DF*_c_ maps are contoured at +3.0*σ* (green) and –3.0*σ* (red). (A) – subunit A, ND_2_; (B) – subunit B, ND_2_; (C) – subunit A, ND^–^, (D) – subunit B, ND^–^. RSZD– values for the N-terminal atom are given for the ND_2_ states to highlight if there are any extra atoms in the model. RSZD+ values for the N-terminal atom are given for the ND^–^ states to highlight if there are any missing atoms in the model.

The structures of the active site resulting from the joint refinement are shown in Fig. 2A and B for subunits A and B, respectively, together with the nuclear density. The coordination environment is the same as in the original crystal structure for both subunits.^28^ Selected structural parameters for the active site are shown in Table 1. The coordination of the dioxygen species is side-on in subunit A (Cu–O distances of 2.18 and 2.48 Å) and end-on in subunit B (Cu–O distances of 1.84 and 2.91 Å), as in the original structure. However, in monomer A, the Cu–O coordination distances are ∼0.3 Å longer than in the original X-ray model (Table 1). This difference can be attributed to the weak nuclear density of the O_2_ unit from the neutron data, especially in subunit A (*cf.*Fig. 2). As oxygen atoms contribute less to the neutron data and our model is refined against both X-ray and neutron data, it is expected that the O_2_ entity will have a different position in joint refinement compared to the neutron-only crystallographic refinement done in ref. 28.

**Table 1:** Cu coordination environment in the various calculations and spin densities of the Cu and oxygen atoms. Distances are in Å^*a*^

Structure	N_ter_	Subu	Cu–N^*ε*^	Cu–N^*δ*^	Cu–N_ter_	Cu–O_prox_	Cu–O_dist_	O–O	H1–O	*q* ^α–β^ Cu	*q* ^α–β^ O_prox_	*q* ^α–β^ O_dist_	Figure
X-ray		A	2.00	2.01	2.12	1.84	2.14	1.48	^*b*^				
X-ray		B	2.01	2.01	2.14	1.83	2.69	1.46	^*b*^				
Neutron	ND_2_	A	2.55	2.56	3.40	^*c*^	^*c*^	^*c*^	^*b*^				
Neutron	ND^–^	B	2.48	2.49	2.94	1.84	2.49	3.37	^*b*^				
JXN-R	ND_2_	A	1.94	1.97	2.12	2.18	2.48	1.45	2.60				Fig. 2A
JXN-R	ND_2_	B	1.92	1.95	2.11	1.84	2.91	1.45	2.41				Fig. 2B
JXN-R	ND^–^	A	1.95	1.97	2.10	2.22	2.52	1.45	2.55				Fig. 2C
JXN-R	ND^–^	B	1.96	1.99	2.10	1.98	2.90	1.45	2.38				Fig. 2D
CQU	ND_2_	A	1.90	1.88	2.19	2.21	2.72	1.35	2.50	0.01	0.48	0.53	Fig. 3A
CQU	ND_2_	B	1.98	1.98	2.18	2.01	2.75	1.38	2.33	0.11	0.53	0.35	Fig. 3B
CQU	ND^–^	A	1.94	1.90	2.17	2.23	2.80	1.37	2.49	0.18	0.40	0.42	Fig. 3C
CQU	ND^–^	B	2.01	1.98	2.15	2.00	2.74	1.39	2.32	0.20	0.46	0.35	Fig. 3D

^*a*^The first column shows the type of calculation (X-ray/neutron for the deposited structures 5VG0 and 5VG1 respectively, JXN-R for joint refinement, CQU for quantum refinement), the second column shows the protonation state of the N-terminus.

^*b*^No deuterium atoms were reported in the deposited X-ray and neutron structure.

^*c*^Subunit A in the deposited neutron structure does not contain any oxygen species bound to copper.

One of the deuterium atoms (D1) in the terminal amino group forms a hydrogen bond to the backbone carbonyl of Ala-107 with an O–D distance of 2.41–2.60 Å. As can be seen in Fig. 2A and B, no nuclear difference-density peaks are observed on the N-terminus at a 3.0*σ* level, neither on subunit A nor on subunit B, indicating that the model with two deuterium atoms is consistent with the crystallographic data. Similarly, no difference density peaks can be seen in the X-ray *mF*_o_ – *DF*_c_ maps (Fig. S2†). This can also be seen in the RSZD– scores of the N-terminus and deuterium atoms, which are <0.1 in both subunits and for both types of maps (X-ray and neutron).

For comparison, we performed also joint refinement of the protein with only one deuterium atom on the N-terminus (*i.e.* a deprotonated amino terminal), both in subunit A and in subunit B. The resulting structures of the active site are shown in Fig. 2C and D, and selected bond distances are given in Table 1. The structures are nearly identical to those in the model with two deuterium atoms, the only atom showing a significant movement is D1, which moves to an intermediate position compared to the two atoms in the previous model (Fig. 2A and B). The remaining deuterium atom still forms a hydrogen bond with the backbone carbonyl of Ala-107. The neutron *mF*_o_ – *DF*_c_ map of the active site of subunit B, which was previously modeled as ND^–^, shows a positive difference density peak at a 3.0–3.2*σ* level, indicating that the ND_2_ state is a better interpretation of the crystal structure, based on the joint refinement. These findings are consistent with the previous joint X-ray–neutron structure of an AA9 LPMO by O'Dell *et al.* (Nc PMO-2, PDB entry ; 5TKI),^27^ which shows no indication of a deprotonated N-terminus.

### Joint X-ray–neutron quantum refinement

Next, we investigated whether the ND_2_ state of the N-terminus agrees both with the crystallographic data and with quantum chemical calculations by quantum refinement. In this approach, we use the X-ray and neutron crystal structure factors and replace the MM potential used in the joint X-ray–neutron refinement by an QM/MM potential. The atoms modeled by a QM potential are shown in Fig. 1.

**Fig. 3:**
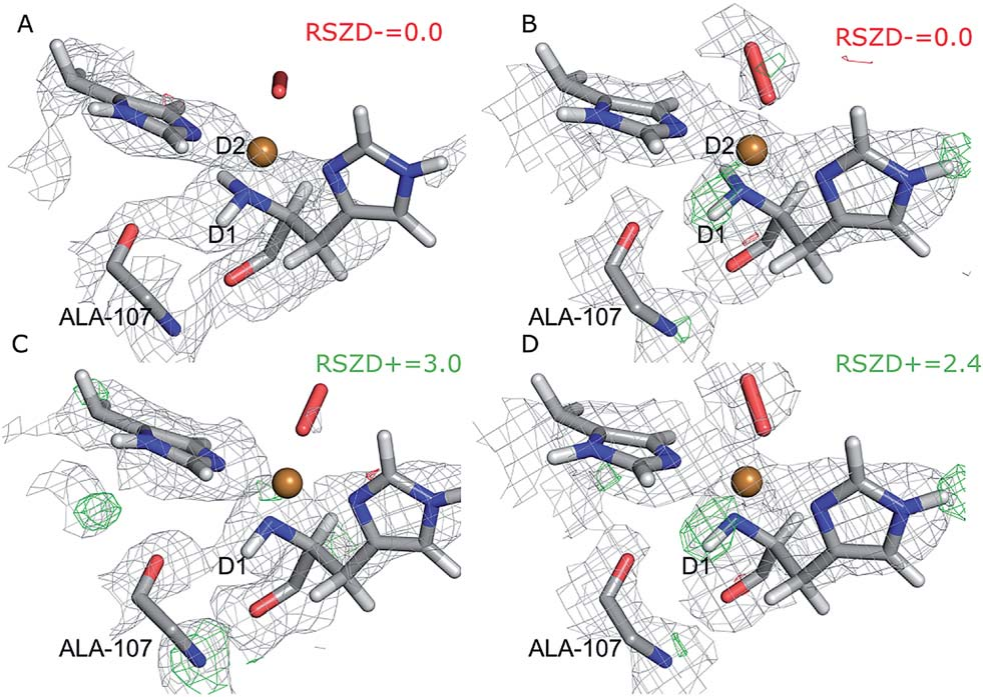
Structure and nuclear density maps of the active site after quantum refinement. *m*2*F*_o_ – *DF*_c_ maps are contoured at 1.0*σ* and *mF*_o_ – *DF*_c_ maps are contoured at +3.0*σ* (green) and –3.0*σ* (red) (A) – subunit A, ND_2_; (B) – subunit B, ND_2_; (C) – subunit A, ND^–^, (D) – subunit B, ND^–^.

The quantum-refined structures are shown in Fig. 3A and B, and selected bond lengths are given in Table 1. The structures are generally in close agreement with the structures obtained by traditional joint X-ray–neutron refinement in both subunits. The D1 atom still forms a hydrogen bond with the backbone carbonyl of Ala-107. The quantum refinement slightly improves the hydrogen bonding geometry, the O–D1 bond length being 0.1 Å shorter than in the structure obtained with traditional joint refinement, 2.50 Å in subunit A and 2.33 Å in subunit B.

Although the density maps calculated from nCNS structures are noisier, the nuclear difference density maps calculated from the quantum-refined structure show no negative density around the D2 atom in either of the subunits. This is also reflected in the RSZD– scores of the N-terminus and the two deuterium atoms, of 1.3–2.2, 0.1–0.5 and 0.8–1.3 respectively, which are all lower than 3.0, albeit higher than in the traditional joint X-ray–neutron refinement. We also performed the quantum refinement with the N-terminus in the ND^–^ state; the resulting structures are shown in Fig. 3C and D. The structures with ND^–^ show no change in the geometry of the active site in either subunit, but a positive nuclear difference density at the 2.4–3.0*σ* level is observed at the position of the D2 atom in both subunits. Thus, the ND_2_ state of the N-terminus agrees better than the ND^–^ state both with the crystallographic data and with quantum-chemical calculations.

Interestingly, the geometry of the oxygen species changes somewhat when a QM potential is introduced. In subunit A, the position of the proximal oxygen atom is identical to the one from joint refinement, whereas the distal oxygen atom moves away from the copper atom, resulting in an end-on coordination with Cu–O_dist_ = 2.72 Å (in contrast to the side-on coordination both in the joint refinement, as well as in the original crystal structure). In subunit B, the dioxygen geometry changes less. The coordination stays end-on, and the Cu–O_prox_ distance is shorter by 0.2 Å.

### Nature and coordination of the oxygen species

While our joint crystallographic and quantum refinement calculations both agree on the protonation state of the N-terminus, the two methods give slightly diverging results concerning the coordination of the dioxygen species. We therefore decided to investigate this issue further, focusing on the structures with two hydrogen atoms on the N-terminus. To this end, we performed QM/MM structure optimisations quantum-refinement calculations, as well as vacuum QM calculations (to calculate strain energies), testing both the CuO_2_, and [CuO_2_]^+^ forms. Considering the low nuclear density for the oxygen atoms, we chose to perform quantum refinement only against the X-ray data.

**Fig. 4:**
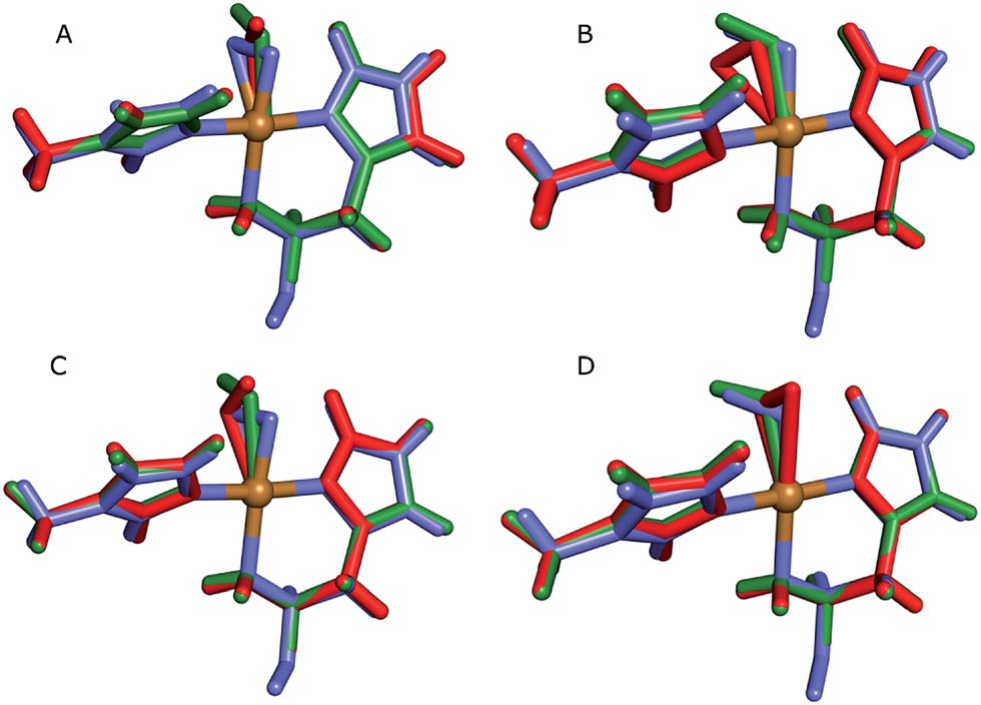
Active sites of the original crystal structure (entry 5VG0) (blue), calculated/quantum-refined structures with peroxide oxygen species (red) or superoxide oxygen species (green). (A) – QM/MM structure, subunit A; (B) – QM/MM structure, subunit B; (C) – quantum-refined structure, subunit A; (D) – quantum-refined structure, subunit B.

Key bonding parameters for Cu and the oxygen species are listed in Table 2, whereas strain energies and RSZD values are summarized in Table 3. The different QM/MM and quantum-refined structures are compared in Fig. 4. A more detailed discussion of the QM/MM and quantum refinement structures is provided in the ESI.† Here we only summarise the main findings.

**Table 2:** Geometric parameters and spin densities of the oxygen species obtained with QM/MM and ComQum-X. For comparison, the original crystal structure is also shown. Distances are given in Å and angles in degrees

Structure	Subunit	State	Cu–O_prox_	Cu–O_dist_	O–O	Cu–O_prox_–O_dist_	*q* ^α–β^ Cu	*q* ^α–β^ O_prox_	*q* ^α–β^ O_dist_	Figure
5VG0	A		1.84	2.14	1.48	79.4				
5VG0	B		1.83	2.69	1.46	109.5				
QM/MM	A	[CuO_2_]	1.99	2.72	1.40	105.8	0.30	0.36	0.24	Fig. 4A
QM/MM conf 1	A	[CuO_2_]^+^	2.08	2.82	1.28	112.0	0.38	0.36	0.16	Fig. 4A
QM/MM conf 2	A	[CuO_2_]^+^	2.02	2.79	1.29	112.9	0.38	0.33	0.20	Fig. S4
QM/MM	B	[CuO_2_]	2.01	2.15	1.40	75.6	0.26	0.44	0.32	Fig. 4B
QM/MM	B	[CuO_2_]^+^	2.06	2.92	1.27	120.2	0.34	0.34	0.15	Fig. 4B
ComQum-X	A	[CuO_2_]	2.21	2.68	1.34	94.8	0.24	0.43	0.37	Fig. 4C
ComQum-X	A	[CuO_2_]^+^	2.31	2.96	1.25	108.6	0.32	0.37	0.21	Fig. 4C
ComQum-X	B	[CuO_2_]	2.20	2.54	1.34	87.8	0.22	0.44	0.37	Fig. 4D
ComQum-X	B	[CuO_2_]^+^	2.27	3.00	1.25	113.6	0.31	0.35	0.22	Fig. 4D
Vacuum		[CuO_2_]	2.03	2.82	1.33	112.5	0.04	0.44	0.53	Fig. S5
Vacuum		[CuO_2_]^+^	2.11	2.88	1.26	115.2	0.29	0.37	0.24	Fig. S5

**Table 3:** Maximum absolute RSZD of the oxygen species and strain energies (Δ*E*_str_) of the system, in kJ mol^–1^, for the structures refined with ComQum-X

Structure	Subunit	State	RSZD_max_	Δ*E*_str_
ComQum-X	A	[CuO_2_]	3.6	68.2
ComQum-X	A	[CuO_2_]^+^	3.7	32.4
ComQum-X	B	[CuO_2_]	6.7	71.3
ComQum-X	B	[CuO_2_]^+^	2.7	31.9

From the vacuum calculations (*cf.*Table 3) we see that the quantum-refined CuO_2_ system is 30 kJ mol^–1^ more strained than the [CuO_2_]^+^ system (for both subunits). Both QM/MM and quantum refinement show that in subunit B, the geometry of the oxygen species in the crystal structure is closer to that of a superoxide ion, as the [CuO_2_]^+^ system has a lower RSZD score and is less strained than the corresponding [CuO_2_] system. In contrast, in subunit A none of the calculations yield geometries that are close to the side-on coordination in the crystal structure and all optimised geometries fit to the data rather poorly. The observed coordination is thus either an artifact or resulting from a superposition of different configurations of the Cu–O_2_ bond. This would also explain why different Cu–O_2_ binding modes are obtained for subunits A and B, and why the density of the oxygen species is so weak in the crystallographic data (see further discussion in the ESI†). To investigate this option, we carried out a potential energy scan of the Cu–O–O angle of the [CuO_2_]^+^ system in vacuum. This investigation reveals that the energy difference between various binding modes of the superoxide is only 15 kJ mol^–1^ (Table S1†). In subunit A, we also obtain two different end-on conformations of [CuO_2_]^+^ (for the peroxide, we always obtained the same conformer). The two conformers are nearly degenerate (within 8 kJ mol^–1^). Therefore, these two conformers may co-exist in the crystal structure (*cf.* Fig. S4†).

### Formation of H_2_O_2_

Knowing the protonation state of the N-terminus and the nature of the oxygen species, we proceeded to study the mechanism of H_2_O_2_ formation.

In a set of initial calculations, we performed QM-cluster calculations of the [CuO_2_]^+^ system and the dissociation products, Cu(ii) and O–2 or Cu(i) and O_2_, respectively. These calculations were carried out at the TPSS/def2-TZVPD level of theory in either gas phase or with a COSMO solvation model (with *ε* = 80). The QM-cluster calculations allow us to obtain zero-point energies (ZPE) and entropies. We have collected the results in Table 4. For both dissociation reactions, the thermochemical corrections (ZPE and entropy) favour dissociation, but their contributions are roughly equal (around 45–55 kJ mol^–1^). The lack of solvation makes dissociation of the oxygen species as superoxide highly disfavoured in gas phase, while dissociation of O_2_ is favourable, mainly owing to the entropy contribution. As expected, solvation has a large stabilizing effect on dissociation of O–2, but the dissociation is still unfavourable by ∼80 kJ mol^–1^, whereas dissociation of dioxygen from Cu(i) is favourable in COSMO solvent with a free energy of –20 kJ mol^–1^.

**Table 4:** Energy components for the dissociation of [CuO_2_]^+^ from QM-cluster calculations in gas phase and COSMO solvent, (*ε* = 80, in kJ mol^–1^. Note that the frequencies are from gas phase calculations and hence ZPVE and entropy effects are estimated from the gas phase

Reaction	Electronic	Solvation	ZPE	–*T*Δ*S*	Δ*G*_vac_	Δ*G*_COSMO_
[CuO_2_]^+^ → Cu(ii) + O–2	920.7	–795.1	–3.9	–42.6	874.3	79.2
[CuO_2_]^+^ → Cu(i) + O_2_	26.0	9.3	–4.8	–50.9	–29.6	–20.3

The above result indicates that dissociation of O–2 is not feasible. In a series of more elaborate QM/MM calculations, we also attempted to dissociate O–2, following the recipe of ref. 26. To this end, we performed several linear transit QM/MM calculations, displacing the O_prox_ atom of the [CuO_2_]^+^ species from the copper atom (up to 3.4 Å), optimising all atoms in the QM system. Similar to the QM-cluster calculations, these calculations were carried out with the def2-TZVPD basis set. During the dissociation, the triplet is always more stable than the (open-shell) singlet. In the equilibrium structure, the singlet–triplet splitting is 15 kJ mol^–1^ and this magnitude is kept at larger distances, although at larger distances the spin density is concentrated on the oxygen atoms, suggesting a Cu(i) state and triplet molecular oxygen.

As also noted in ref. 26, the use of a triple-zeta basis set has some influence and results in an equilibrium structure where the axial water molecule no longer binds to the copper (the Cu–OH_2_ distance is 2.83 Å), in contrast to the calculations in the previous section, which were done with the def2-SV(P) basis set. At a Cu–O_prox_ distance of 3.0 Å the water molecule binds back to the copper atom in the axial position, as shown in Fig. S7.† This is again consistent with the findings of Kjaergaard *et al.*^26^. However, unlike ref. 26, we did not observe any transfer of a proton from the water ligand to the oxygen ligand, even if we changed the orientation of the water molecule to be optimal for proton transfer. Moreover, if the restraints on the Cu–O_prox_ distance were removed, the O_2_ entity binds back to the copper atom, forming the [CuO_2_]^+^ species again. This is perhaps understandable given the results in vacuum where dissociation is strongly disfavored, even with inclusion of solvent (*cf.*Table 3).

In an attempt to keep the oxygen fully dissociated, we included six additional water molecules in the QM system and reoptimised from a structure with the O_2_ already dissociated, at a Cu–O_prox_ distance of 4.6 Å, without any restraints (Fig. S8A†). However, the optimisation also led to a structure with the superoxide bound to the copper atom, suggesting that the release of the unprotonated superoxide does not occur.

Prompted by our previous study on an AA9 LPMO^21^ we next investigated a mechanism in which [CuO_2_]^+^ is successively protonated and reduced. We investigated the following six reactions:8
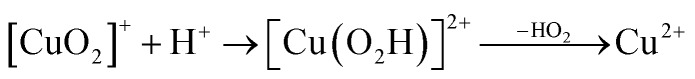

9 [Cu(O_2_H)]^2+^ + H^+^ → [Cu(O_2_H_2_)]^3+^

10
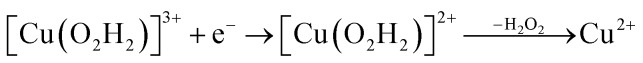

11
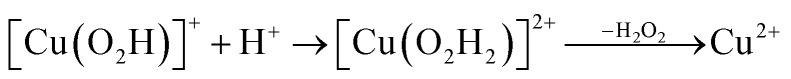

12
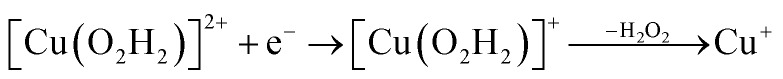

13
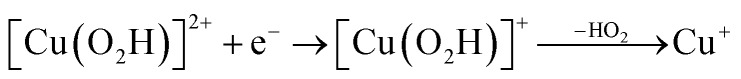
where dissociation of the dioxygen species, HO_2_ or H_2_O_2_, may occur from either Cu(ii) in [Cu(O_2_H)]^2+^ or [Cu(O_2_H_2_)]^2+^ or from Cu(i) in [Cu(O_2_H)]^+^ or [Cu(O_2_H_2_)]^+^.

**Fig. 5:**
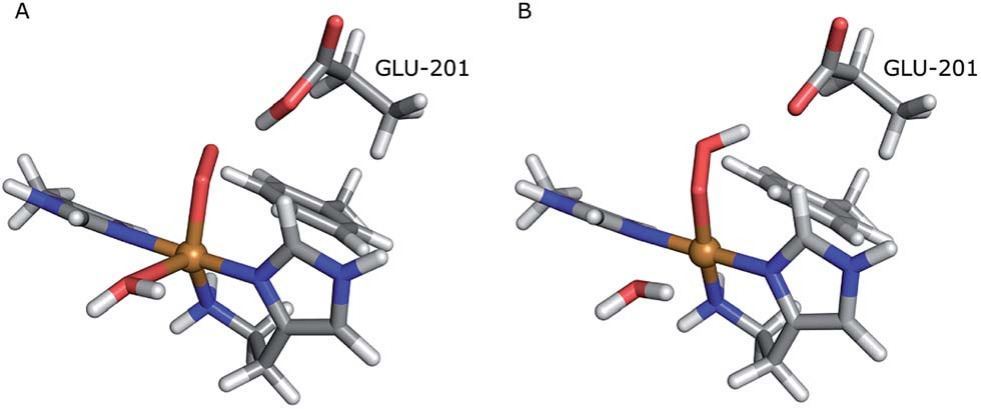
Protonation of the superoxide moiety. (A) – reactant, proton on Glu-201 (triplet); (B) – product with the superoxide protonated (open-shell singlet).

The first protonation of the superoxide species (eqn (8)) was started from the previously optimised equilibrium structure of [CuO_2_]^+^, with a proton added to the Glu-201 residue (*cf.*Fig. 5A). In the triplet spin state, the proton from Glu-201 does not transfer to O–2 and all attempts to start from the product (with Cu–O_2_H) leads back to the reactant. In contrast, for the open-shell singlet the Glu-201 proton transferred spontaneously to the distal oxygen of the superoxide, resulting in a Cu–O_2_H species (Fig. 5B), with a Cu–O_prox_ distance of 1.88 Å, 0.05 Å shorter than with the unprotonated O–2. The reaction energy of this transfer of the proton from Glu-201 to the superoxide moiety is –8 kJ mol^–1^.

**Fig. 6:**
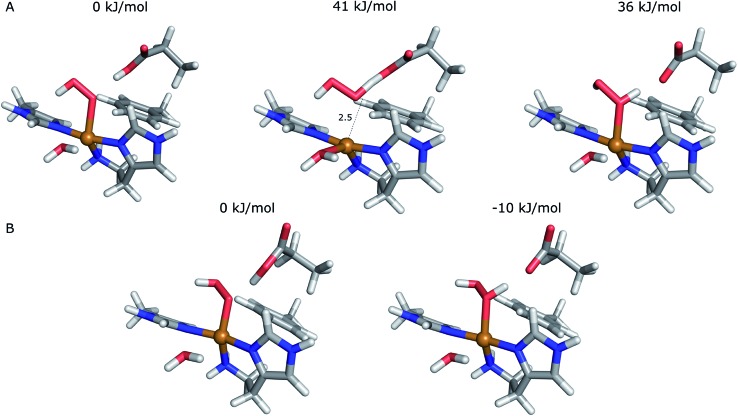
Second protonation of the superoxide moiety starting from (A) – Cu(ii)–HO_2_ or (B) – Cu(i)–O_2_H. Relative energies of the reactants, products and transition state (for Cu(ii)–O_2_H) are depicted above each structure.

Starting from the [Cu(O_2_H)]^2+^ species, we performed a second protonation, resulting in the [Cu(O_2_H_2_)]^3+^ species (eqn (9)). The proton was moved in 0.1 Å steps from the OE1 atom of the glutamate to the proximal oxygen atom of the superoxide. The reaction energy for this proton transfer was 36 kJ mol^–1^, with a barrier of 41 kJ mol^–1^. Interestingly, this second protonation occurs with an un- and re-binding of HO_2_, the superoxide species moving as far as 2.4 Å away from the copper ion in the transition state, as shown in Fig. 6A. However, if we attempt the second protonation after first reducing the copper center to Cu(i) (first part of eqn (11) and (13)), the proton transfers practically spontaneously from the Glu-201 to the HO_2_ species (the barrier is 1 kJ mol^–1^). Furthermore, the reaction energy is –10 kJ mol^–1^, showing that this protonation pathway is favoured (or the reaction is a concerted proton-electron transfer).

Next, we investigated the release of HO_2_ or H_2_O_2_ through linear-transit calculations. The dissociation turned out not to be possible from Cu(ii) as in eqn (8), (10) and (11); unrestrained optimisation starting from any of the investigated Cu–O_prox_ distances (up to 3.4 Å) always led back to the starting structures of [Cu(O_2_H)]^2+^ or [Cu(O_2_H_2_)]^2+^, as found for the unprotonated superoxide. Once again, we also extended the quantum system with six water molecules in order to optimise the water hydrogen bonding network around HO_2_ or H_2_O_2_ in attempt to keep the two molecules dissociated. Yet, even starting with the protonated dioxygen moieties at a distance of 4.6 Å to the copper atom (Fig. S8†), both HO_2_ and H_2_O_2_ bind back to the copper atom in unrestrained optimisations, the final geometries being nearly identical to the starting equilibrium states.

**Fig. 7:**
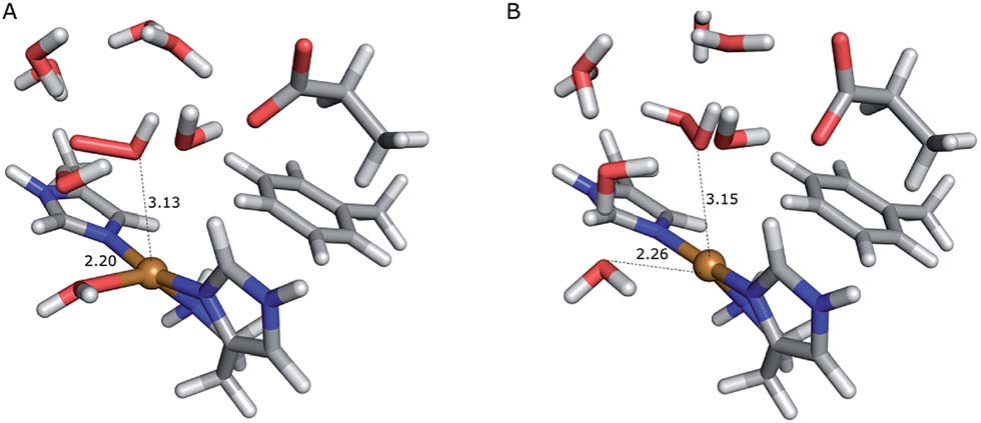
Release of the dioxygen moiety after reduction of the copper center to Cu(i). (A) – optimised Cu(i)–HO_2_ system; (B) – optimised Cu(i)–H_2_O_2_ system.

On the other hand, release of the dioxygen moiety was possible after reducing the copper center to Cu(i) in accordance with eqn (12) and (13). Optimisations of dissociated HO_2_ or H_2_O_2_ (still including six water molecules in the quantum system) resulted in stable structures with the dioxygen moieties released from the active site, at Cu–O distances of 3.13 and 3.15 Å, respectively (Fig. 7). The spin density on the dissociated HO_2_ is ∼0.8, showing that the system indeed contains Cu(i) and HO_2_, even at larger Cu–HO_2_ distances. Interestingly, the resulting Cu(i) coordination sphere is different in the HO_2_ calculation compared to the H_2_O_2_ calculation. In the former, the Cu(i) adopts a trigonal pyramid geometry, with a water molecule in the axial position (Cu–O_wat_ distance of 2.2 Å), whereas in the latter, the copper ion is only coordinated by the three histidine nitrogen atoms, in a trigonal geometry, the Cu–O_wat_ distance becoming 2.26 Å.

For Cu(i), linear-transit calculations between the equilibrium structures and the structures with the dioxygen species dissociated show that dissociation is much easier for H_2_O_2_ than for HO_2_. The dissociation of HO_2_ has a barrier of 60 kJ mol^–1^, whereas H_2_O_2_ dissociates from Cu(i) with a barrier of only 4 kJ mol^–1^. Moreover, the reaction energy for the dissociation of HO_2_ is higher than that for the dissociation of H_2_O_2_, 28 kJ mol^–1^ compared to 3 kJ mol^–1^. However, assuming entropy and ZPVE contributions of similar size as for the dissociation of O_2_ or O–2, HO_2_ dissociation would be roughly thermoneutral.

Therefore, we conclude that the formation of the hydrogen peroxide co-substrate in AA10-LPMO is possible only after the reduction of the active site by one electron. Hydrogen peroxide could be formed from the release and disproportionation of HO_2_, but the dissociation energy of HO_2_ is higher than that of H_2_O_2_. Since protonation of HO_2_ (after reduction of the active site) is practically spontaneous, and dissociation of H_2_O_2_ has a very low activation energy this seems to be the preferred pathway. Our favoured overall pathway involves Cu(i) and consumes two protons and two electrons leading to H_2_O_2_ and regeneration of Cu(i), corresponding to eqn (2), first parts of eqn (8) and (13) and eqn (11) and a total catalytic reaction14
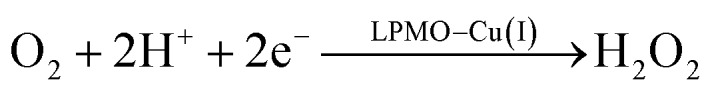



In all cases the proton donor is Glu-201. The steps are either spontaneous or involve barriers lower than 10 kJ mol^–1^. We note that while this pathway gives the lowest activation energies, the pathways involving eqn (9) and last part of 13 have barriers of 41 and 60 kJ mol^–1^ (where the latter should be considered an upper limit as it does not include thermochemical corrections). These are both feasible, considering that the reported lower-limit rate constant^26^ is 0.15 s^–1^, corresponding to an activation energy of ∼80 kJ mol^–1^, as calculated from transition state theory. In summary, we cannot find support for dissociation of neither O–2, HO_2_ nor H_2_O_2_ from Cu(ii). However, alternate protonations and reductions starting from [CuO_2_]^+^ with Glu-201 as proton donor, followed by dissociation of either HO_2_ or (more likely) H_2_O_2_ from Cu(i) are feasible. This is different than the hitherto accepted molecular mechanism^26^ involving direct release of O–2. In light of our results, we speculate that the formation of H_2_O_2_ from release of O–2 only becomes relevant if the proton donors in the second coordination sphere are perturbed, *e.g.*, by mutations. Such a study was recently performed by Span *et al.*^54^ (albeit on a different LPMO, see further below). Their study indeed indicated H_2_O_2_ formation from O–2 in solution increased by such mutations. While we here have only discussed one specific AA10 LPMO, it is naturally interesting to assess how general our suggested mechanism is for other LPMO classes. The large sequence variation among the LPMOs makes such an assessment difficult. However, a general idea can be obtained by comparing the second coordination sphere for a few different LPMOs. Among the AA10 LPMOs, there is not only variation compared to AA9, but also considerable variation within the group, depending on their substrate specificity (chitin or cellulose).^55^ The target LPMO in this study is JdLPMO10A, whose substrate is chitin;^56^ cellulose-active AA10 LPMOs also have conserved hydrogen-bonding motifs in the second coordination sphere.^5^,^57^ For example, the structure with PDB entry ; 4OY7 (ref. 57) has both an Arg and a Glu residue (Arg-212 and Glu-217) close to the Cu ion.^57^ These residues could play the same role as Glu-201 in our calculations. In AA9 LPMOs, a histidine and a glutamine (His-147 and Gln-162 in ; 5ACF 31) replace the Arg and Glu residues from cellulose-active AA10 LPMOs and these two residues are generally conserved among AA9 LPMOs. Proton transfer from His147 to [CuO_2_]^+^ has been found to be feasible,^21^ making it likely that the current mechanism is also valid for AA9 LPMOs. Yet, further investigations are required before we can truly comment on how general our current mechanism is.

## 4 Conclusions

In this article, we have studied the mechanism of hydrogen peroxide generation from a recent AA10 LPMO structure with a bound dioxygen species.^28^ Since the reduction level and protonation state of this species is ambiguous, we have first employed quantum refinement of the crystal structure against both X-ray and neutron data to obtain a proper description of the active site. These calculations show conclusively that the N-terminus, which coordinates to the copper atom, is not deprotonated in the crystal structure. Furthermore, both quantum-refinement and QM/MM calculations indicate that the dioxygen species bound in the crystal is a superoxide, O–2, which is in accordance to the study by Kjaergaard *et al.*^26^ on an AA9 LPMO. The mechanism of H_2_O_2_ generation was studied using the QM/MM approach, at the TPSS/def2-TZVPD level. The results show that unprotonated superoxide does not dissociate after protonation from a water molecule, as seen in the AA9 LPMO studied by Kjaergaard *et al.*^26^ Moreover, we were unable to dissociate any dioxygen species from Cu(ii), no matter the protonation and reduction state. However, dissociation of both HO_2_ and H_2_O_2_ is possible from Cu(i); the former can then generate hydrogen peroxide in bulk through disproportionation. The QM/MM calculated energies show that dissociation of H_2_O_2_ from Cu(i) is more favourable than that of HO_2_. Thus, our proposed mechanism consists of adding two protons and two electrons to dissociate hydrogen peroxide from Cu(i). The first protonation is spontaneous, whereas the second protonation has a very low activation barrier, provided that the first electron has been added to the system. Then, hydrogen peroxide dissociates from Cu(i) with a barrier of only 4 kJ mol^–1^, in contrast to hydrogen superoxide, which needs to overcome a dissociation energy barrier of 60 kJ mol^–1^. In summary, our calculations support that hydrogen peroxide needs to be formed at a Cu metal center through successive protonations and reductions before being dissociated into the bulk to act as a co-substrate for the monooxygenase reaction.

## Conflicts of interest

There are no conflicts to declare.

## Supplementary Material

Supplementary information
