# Supplementary material for: Mechanism of hydrogen peroxide formation by lytic polysaccharide monooxygenase
Source: Chem Sci. 2018 Oct 19;10(2):576–86. doi: 10.1039/c8sc03980a (PMC6334667; doi:10.1039/c8sc03980a)
Supplement: Supplementary file 1 [file SC-010-C8SC03980A-s001.pdf]

Electronic Supplementary Material (ESI) for Chemical Science.
This journal is © The Royal Society of Chemistry 2018

Supporting Information

Mechanism of hydrogen peroxide formation by lytic polysaccharide monooxygenase

Octav Caldararu,[a], Esko Oksanen,[bc], Ulf Ryde[a], Erik D. Hedegård*[a]

[a] Division of Theoretical Chemistry, Lund University, Chemical Centre, P. O. Box 124, SE-221 00 Lund
erik.hedegard@teokem.lu.se
[b] European Spallation Source ESS ERIC, P. O. Box 176, SE-221 00 Lund, Sweden [c] Department of Biochemistry and Structural Biology, Lund University, Chemical Centre, P. O. Box 124, SE-221 00 Lund, Sweden

Protein setup

The starting coordinates for QM/MM calculations were taken from the joint refinement reported in the main paper. The structure is a dimer that contains 288 amino acids, 299 crystal water molecules, amounting to 5105 atoms in total.

The crystal structure contained 203 amino-acid residues and 105 water residues with atoms in alternative conformations. We included in the calculations only the conformation with highest occupation or the first conformation if the occupation numbers were equal. Hydrogen atom coordinates were taken from the joint refinement reported in the main paper. The individual residues were additionally visually inspected and their solvent exposure and hydrogen-bond network were assessed. Based on this, we concluded that all arginines and lysines are protonated (+1) and the aspartic and glutamic acids are in their carboxylate form (−1). In both chains, Arg41 and Arg154 forms ionic pairs with the carboxylate groups of Asp169 and Glu115, while His149 (doubly protonated, see below) forms an ionic pair with Asp119. In one case, a ionic pair is formed between the two chains: The ammonium group of Lys68 in chain A forms a ionic pair with the carboxylate group of Glu60 in chain B.

The protein contains four cysteine residues in each chain (Cys45, Cys54, Cys73 and Cys167) that are cross-linked by disulfide bridges. Residues 1-31 were missing and no attempt to build these missing residues was done.

Concerning the histidine residues, these can have two possible protonation sites. The dimer has 5 histidine residues in each chain. In the following, we denote histidines as HIE ($N^{\varepsilon 2}$ protonated), HID ($N^{\delta 1}$ protonated) or HIP (both nitrogens protonated). The N-terminal histidine is a special case since $N^{\delta 1}$ (and the terminal N) coordinates to the Cu ion. In some LPMOs, the imidazole ring of the terminal histidine is methylated on the $N^{\varepsilon 2}$ atom, but this is not the case for the one studied here. For the remaining histidine residues, we employed the protonation states HID109, HIP123, HIP139 and HIP149. HID109 coordinates to Cu through $N^{\varepsilon 2}$. The two residues HIP123 and HIP139 are close to the surface, and therefore chosen to be doubly protonated. The last histidine, HIP149, is also on the surface and forms additionally hydrogen bonds to the carbonyl O of Thr115 through $N^{\varepsilon 2}$, and to the carboxylate $O^{\delta 2}$ of Asp119 through $N^{\delta 1}$ (cf. description of ionic pairs above). The total charge of the simulated system in the $[CuO_2]^+$ state was -12 (for the dimer).

Equilibration and QM/MM setup

The system described above was equilibrated by simulated annealing. Both the equilibration and the QM/MM calculations followed closely our previous investigations[1,2]. Hence, the protein was described with the Amber FF14SB force field[3] and water molecules with the TIP3P model.[4]

For the equilibration, restrained electrostatic potential (RESP) charges were employed for the metal center and its first coordination sphere (cf. Figure S1). The employed structure geometry optimised, employing the TPSS functional[5] together with the def2-SV(P) basis set[6,7]. The electrostatic potential was calculated in points that were sampled with the Merz–Kollman scheme[8,9] using default radii for the light atoms and 2 Å for Cu[10]. They were employed by the RESP program (a part of the AMBER software package) to calculate the charges.

As described in the main paper, the QM/MM structure optimizations employed the dispersion-corrected TPSS-D3 functional[5,11] with Becke–Johnson damping[12] and the def2-SV(P) or def2-TZVPD basis sets[6,7].

QM/MM and QM-refinement of oxygen-bound X-ray structure

Table S1 Energies of the $[CuO_2]^+$ system at Cu–O–O angles varying from 80° to 150°. Energies are calculated relative to the minimum energy (at 110°). Angles are given in degrees and energies in kJ/mol.

Cu–O–O angle	ΔE
80	2.38
90	0.36
100	0.14
110	0.00
120	7.75
130	15.65
140	5.38
150	2.11

In both subunits, the triplet state of the $[CuO_2]^+$ system was ∼15 kJ/mol lower in energy than the open-shell singlet and the active site geometry shows no significant differences between the two spin states. Therefore, we discuss only results obtained in the triplet state.

Analysis of the electronic structure through examination of the spin density reveals that for $[CuO_2]^+$, the spin density is located both on the Cu atom and on the O_2 entity, approximately 0.38 on the copper atom and each of the oxygen atoms, suggesting a Cu(II)–$O_2{}^-$ state. Moving to the CuO_2 state changes the spin density on Cu with only 0.08, suggesting that Cu remains Cu(II) and the species is best described as Cu(II)–O_2^{2-}, i.e., a peroxide state.

We start by discussing the results of the QM/MM calculations. For subunit A, we were not able to obtain any side-on bound oxygen species: both peroxide and superoxide coordinate end-on to the copper, unlike in the crystal structure, and in fairly different positions, with a 26° larger Cu–O–O angle (Table 2 and Figure 4A). In subunit B, QM/MM calculations for the neutral CuO_2 system yields a side-on coordination of the peroxide to the copper atom, unlike in the original crystal structure. In contrast, the $[CuO_2]^+$ system shows an end-on binding of the superoxide moiety (with Cu–O_{dist} = 2.15 Å), as observed in the crystal structure.

The quantum-refined structure of subunit A (cf. Figure 4C) suggests end-on coordination for both the peroxide and superoxide complexes. This indicates that the QM energy component in Eq. (1) favours O_2 binding in an end-on fashion. This is confirmed by unrestrained optimisations of the QM system in vacuum, which give rise only to the end-on binding mode (also included in Table 2). As expected, the oxygen atom positions are closer to the original crystal structure than to the QM/MM minimized structure. Although both peroxide and superoxide bind to copper at similar distances, the proximal oxygen position is different in the two cases. In the peroxide structure, the proximal oxygen atom occupies a position out of the plane formed by the three coordinating atoms. In contrast, the superoxide binds to the copper close to the equatorial plane, in a position similar to where the proximal oxygen atom lies in the B subunit. The RSZD scores of both $[CuO_2]^+$ and CuO_2 systems are over 3.0 and the $mF_o - DF_c$ maps show positive difference density around the proximal oxygen atom (Figures S3A and B), implying that neither structure fits the data well. This once again suggests that the observed oxygen binding in subunit A might be a superposition of different conformations and is not well represented by a single structure.

In subunit B, $[CuO_2]^+$ gives rise to an end-on conformation, as in the original crystal structure, whereas the neutral CuO_2 system shows a side-on conformation with the Cu–O_{dist} bond 0.3 Å longer than the Cu–O_{prox} bond. The quantum-refined structure with $[CuO_2]^+$ is closer to the original crystal structure, whereas in the neutral $[CuO_2]$ system, dioxygen binds with the distal oxygen atom pointing in the opposite direction to that in the crystal structure (Figure 4D). Furthermore, the RSZD score of the oxygen species in subunit B is much lower for the superoxide than for the peroxide (2.7 compared to 6.7), showing that the superoxide fits the X-ray data better. This can also be seen in the $mF_o - DF_c$ maps, as the peroxide structure shows negative difference density around the proximal oxygen atom, which is not present in the structure containing a superoxide ion (Figures S3C and D).

It is interesting to note that the quantum refinements give a Cu–O_{prox} distance that is 0.2 Å longer than that resulted from pure QM/MM. This suggests that other species binding more weakly to copper (e.g. H_2O) could exist in the crystal structure. However, quantum refinement with a water molecule in subunit A instead of a dioxygen species gives rise to a positive difference density where the second oxygen atom should be (Figure S6), showing that a dioxygen species is

Table S2 Mulliken partial charges of important atoms in the oxygen species obtained with QM/MM and ComQum-X.

Structure	Subunit	State	q Cu	q O_{prox}	q O_{dist}
ComQum-U	A	$[CuO_2]$ ND_2	0.29	-0.29	-0.31
ComQum-U	A	$[CuO_2]$ ND^-	0.32	-0.37	-0.35
ComQum-U	B	$[CuO_2]$ ND_2	0.33	-0.34	-0.35
ComQum-U	B	$[CuO_2]$ ND^-	0.31	-0.38	-0.35
QM/MM	A	$[CuO_2]$	0.36	-0.31	-0.44
QM/MM conf 1	A	$[CuO_2]^+$	0.46	-0.12	-0.21
QM/MM conf 2	A	$[CuO_2]^+$	0.45	-0.14	-0.23
QM/MM	B	$[CuO_2]$	0.31	-0.25	-0.26
QM/MM	B	$[CuO_2]^+$	0.42	-0.12	-0.15
ComQum-X	A	$[CuO_2]$	0.28	-0.28	-0.30
ComQum-X	A	$[CuO_2]^+$	0.45	-0.12	-0.22
ComQum-X	B	$[CuO_2]$	0.30	-0.30	-0.30
ComQum-X	B	$[CuO_2]^+$	0.43	-0.11	-0.17
Vacuum		$[CuO_2]$	0.30	-0.27	-0.27
Vacuum		$[CuO_2]^+$	0.45	-0.09	-0.13

bound to copper in the crystal. Still, this does not exclude the possibility that a water molecule could have replaced the dioxygen species in some unit cells, causing the longer Cu–O_{prox} we observe in the refinements.

Notes and references

1 E. D. Hedegård and U. Ryde, *ACS Omega*, 2017, **2**, 536–545 (arXiv:1612.05513).

2 E. D. Hedegård and U. Ryde, *Chem. Sci.*, 2018, **9**, 3866–3880.

3 J. A. Maier, C. Martinez, K. Kasavajhala, L. Wickstrom, K. E. Hauser and C. Simmerling, *Journal of Chemical Theory and Computation*, 2015, **11**, 3696–3713.

4 P. Jørgensen, P. Swanstrøm and D. L. Yeager, *J. Chem. Phys.*, 1983, **78**, 347–356.

5 J. Tao, J. P. Perdew, V. N. Staroverov and G. E. Scuseria, *Phys. Rev. Lett.*, 2003, **91**, 146401.

6 A. Schäfer, H. Horn and R. Ahlrichs, *J. Chem. Phys.*, 1992, **97**, 2571–2577.

7 K. Eichkorn, F. Weigend, O. Treutler and R. Ahlrichs, *Theor. Chem. Acc.*, 1997, **97**, 119–124.

8 C. A. Singh and P. A. Kollman, *J. Comp. Chem.*, 1984, **5**, 129–45.

9 B. H. Besler, K. M. Merz Jr. and P. A. Kollman, *J. Comp. Chem.*, 1990, **11**, 431–439.

10 E. Sigfridsson and U. Ryde, *J. Comp. Chem.*, 1998, **19**, 377–395.

11 S. Grimme, J. Antony, S. Ehrlich and H. Krieg, *J Chem. Phys.*, 2010, **132**, 154104.

12 S. Grimme, S. Ehrlich and L. Goerigk, *J. Comput. Chem.*, 2011, 1456–1465.

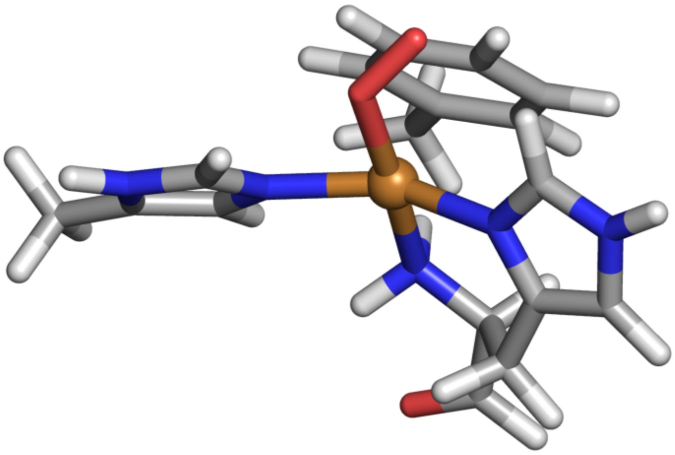

Figure S1 The system employed for calculating RESP charges.

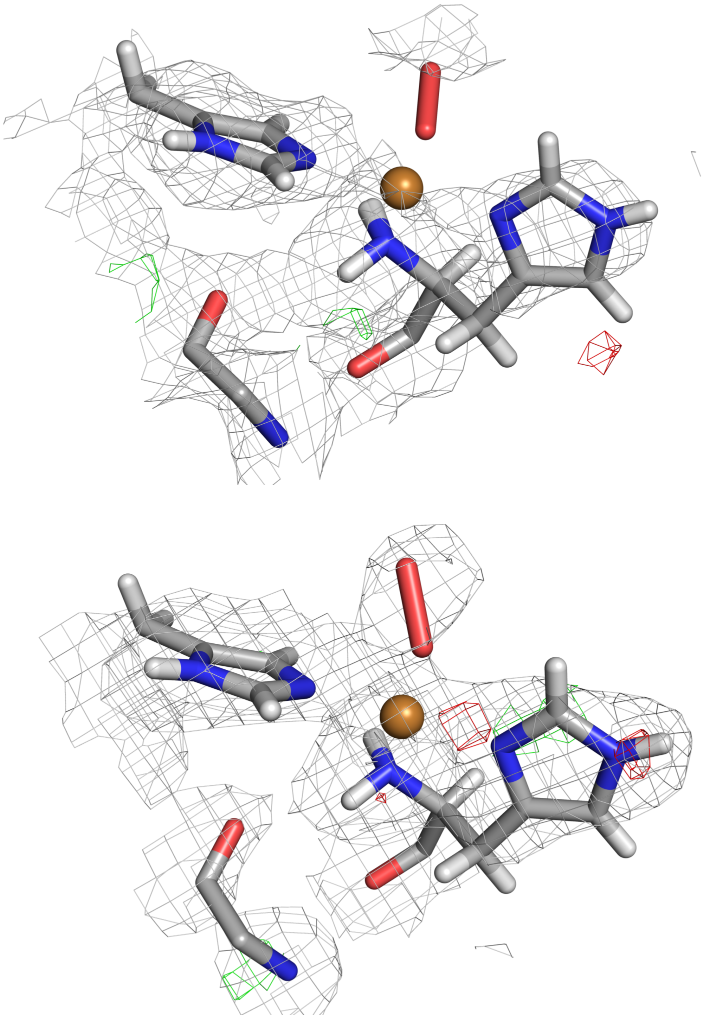

Figure S2 Structure and electron density maps of the active site after joint refinement. The $m2F_o - DF_c$ map is contoured at 1.0σ and the $mF_o - DF_c$ maps are contoured at +3.0σ (green) and -3.0σ (red). Top - subunit A ; Bottom - subunit B

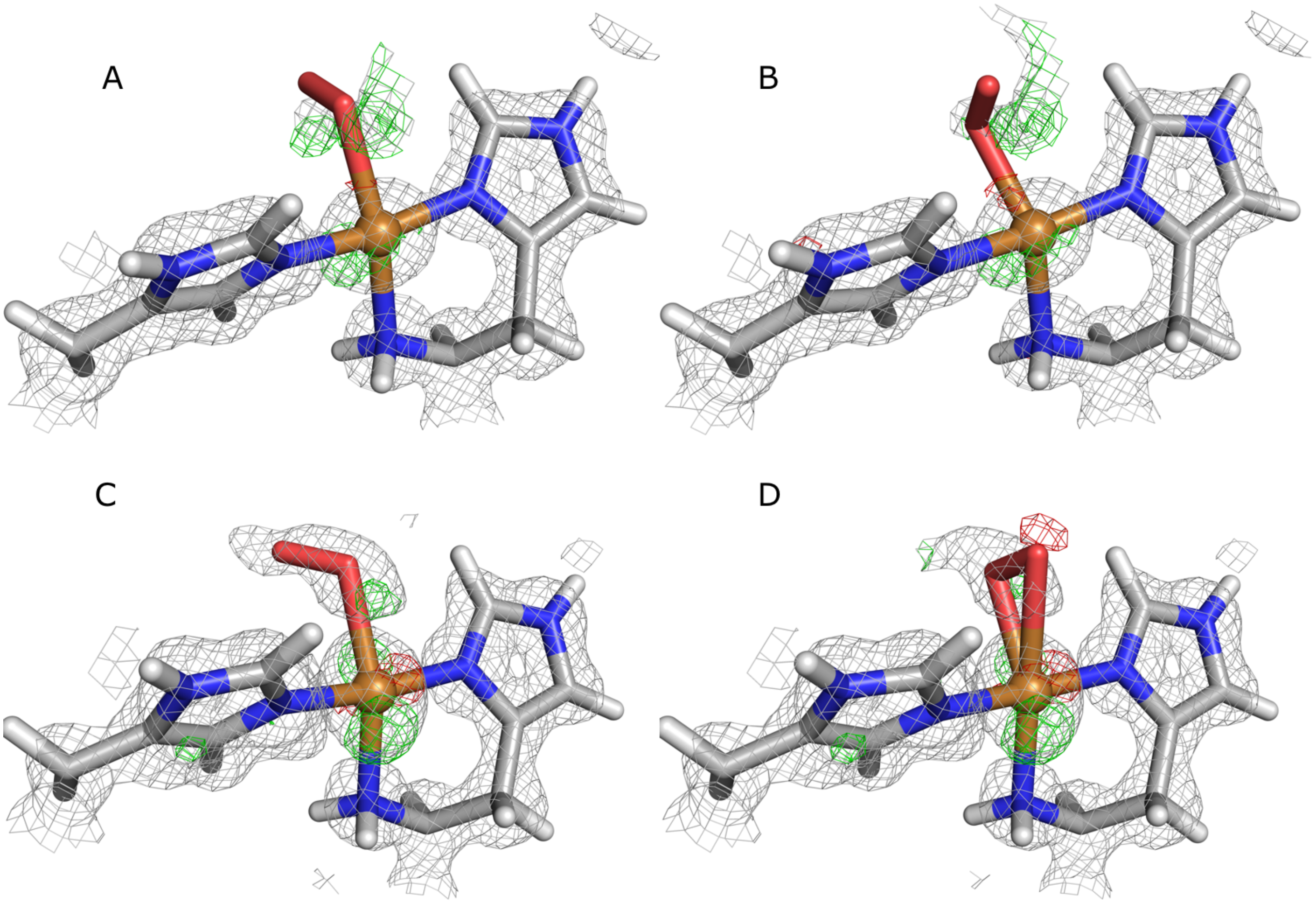

Figure S3 Structure and electron density maps of the active site obtained from quantum refinement against the X-ray data. $m2F_o - DF_c$ map is contoured at 1.0σ and $mF_o - DF_c$ maps contoured at +3.0σ (green) and -3.0σ (red) A - subunit A, superoxide; B - subunit A, peroxide; C - subunit B, superoxide; D - subunit B, peroxide.

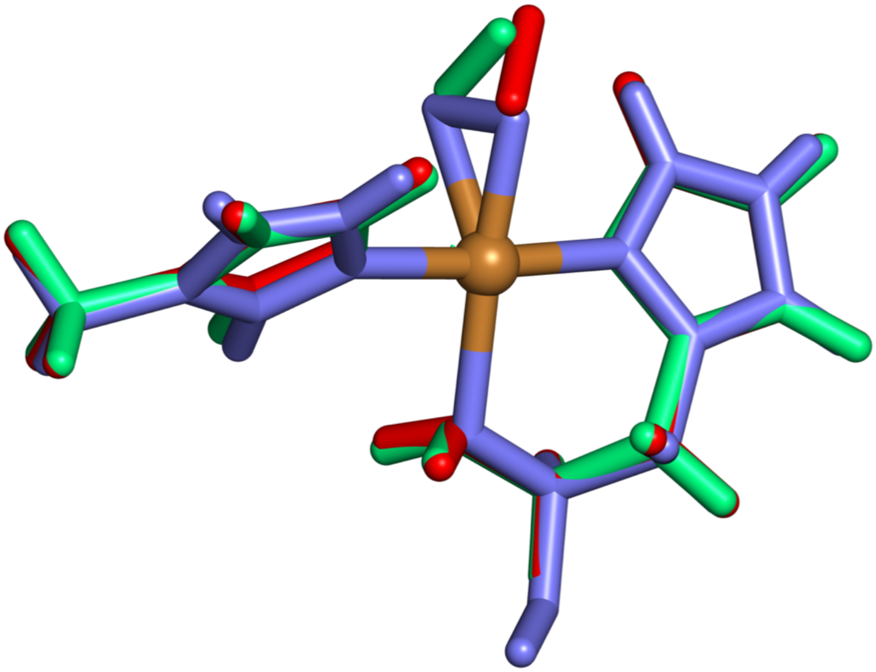

Figure S4 Active sites of the original crystal structure (entry 5VG0) (blue) and two QM/MM minimised structures with a superoxide oxygen species starting from different structures (green and red).

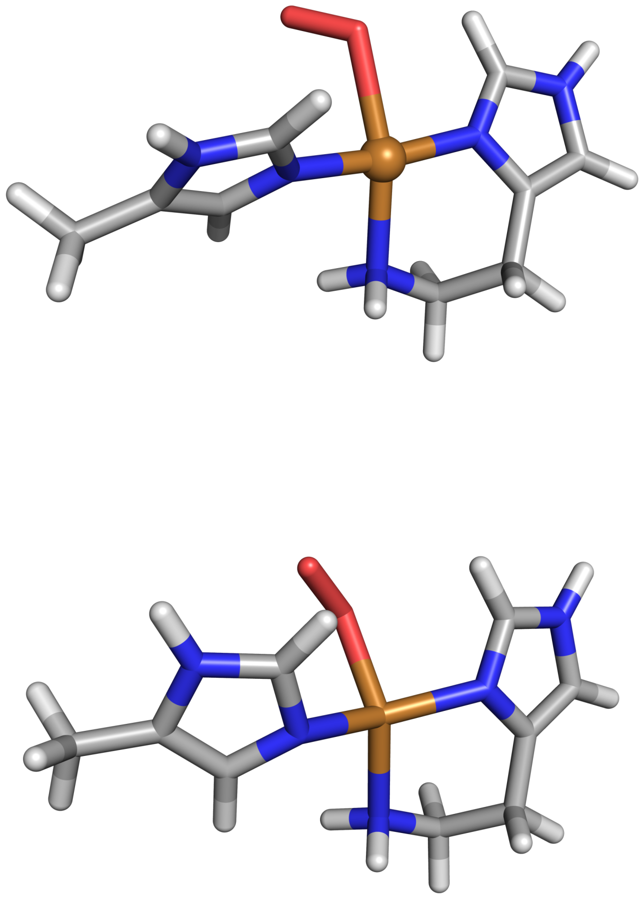

Figure S5 Active sites of the AA10-LPMO optimised in vacuum, resulting in end-on binding mode of the di-oxygen species. Top - $[CuO_2]^+$ Bottom - $[CuO_2]$

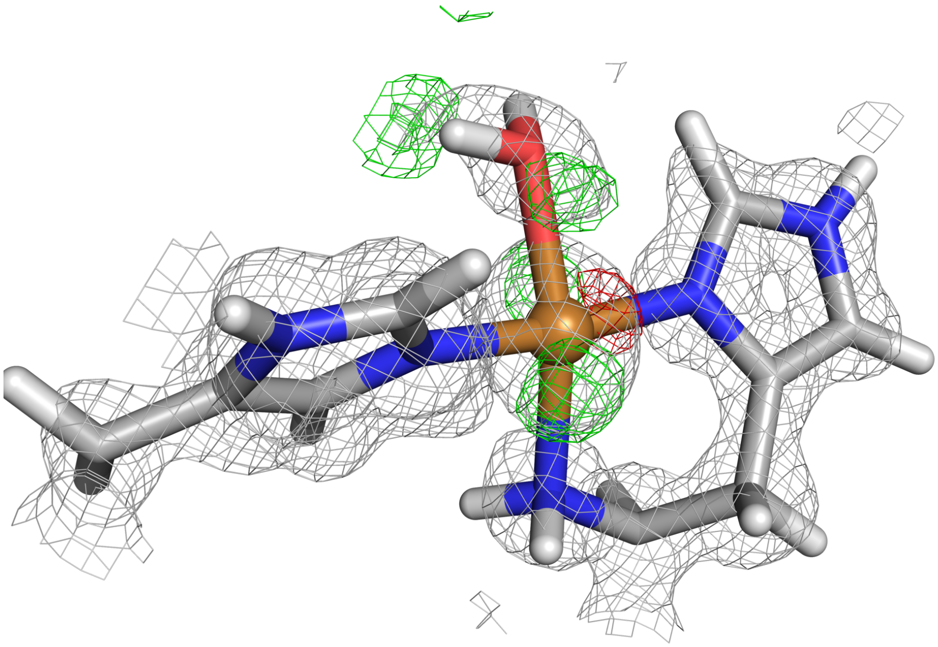

Figure S6 Structure and electron density maps of the active site with a water molecule replacing the di-oxygen species after quantum refinement against X-ray data. The $m2F_o - DF_c$ map is contoured at 1.0σ and the $mF_o - DF_c$ maps are contoured at +3.0σ (green) and -3.0σ (red).

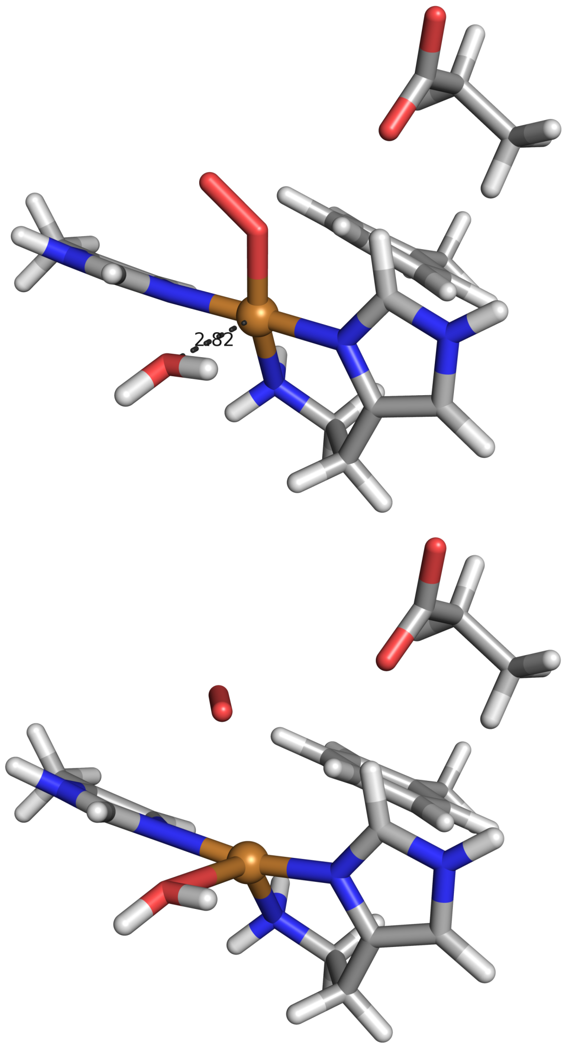

Figure S7 Release of the unprotonated superoxide moiety. Top - no restraints, water molecule not bound to Cu; Bottom - 3.0 Ådistance restraints between Cu and O, water molecule bound to Cu.

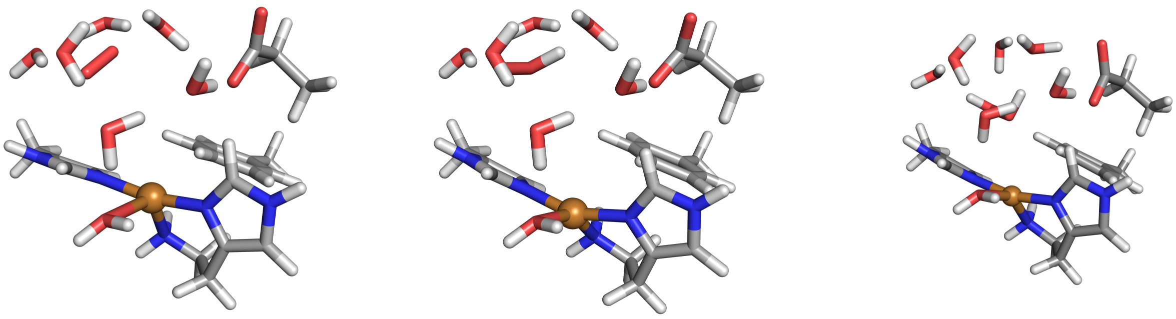

Figure S8 Extended Cu(II) system with 6 water molecules and Right - O_2^-, Middle - HO_2, Left - H_2O_2.
